# Efficacy and Safety of Polaprezinc (Zinc Compound) on Zinc Deficiency: A Systematic Review and Dose–Response Meta-Analysis of Randomized Clinical Trials Using Individual Patient Data

**DOI:** 10.3390/nu12041128

**Published:** 2020-04-17

**Authors:** Kei Furihata, Masaru Tsuchikawa, Takaki Miwa, Yuji Naito, Koji Oba, Masafumi Sakagami

**Affiliations:** 1 Biostatistics, Data Science, Clinical Administration, Zeria Pharmaceutical Co., Ltd., 10-11, Nihonbashi Kobuna-cho, Chuo-ku, Tokyo 103-8351, Japan; 2 Department of Otorhinolaryngology, Kanazawa Medical University, 1-1 Daigaku, Uchinada, Ishikawa 920-0293, Japan; 3 Department of Molecular Gastroenterology and Hepatology, Kyoto Prefectural University of Medicine, Graduate School of Medical Science, 465 Kajii-chi, Kawaramachi-Hirokoji Kamigyo-ku, Kyoto 602-8566, Japan; 4 Department of Biostatistics, School of Public Health, Graduate School of Medicine, The University of Tokyo, 7-3-1, Hongo, Bunkyo-ku, Tokyo 113-0033, Japan; 5 Department of Otolaryngology-Head and Neck Surgery, Hyogo College of Medicine, 1-1 Mukogawa, Nishinomiya, Hyogo 663-8501, Japan

**Keywords:** systematic review, IPD meta-analysis, zinc deficiency, polaprezinc, zinc L-carnosine, serum zinc concentration

## Abstract

Zinc intake is recommended for zinc deficiency. In clinical practice, polaprezinc has been used as a zinc replacement therapy for zinc deficiency. However, the efficacy of polaprezinc has not been established. To confirm the efficacy on zinc deficiency of polaprezinc and provide additional information on an appropriate regimen, we conducted a systematic review using individual patient data (IPD). We searched PubMed, the Japanese database Ichushi, and the database owned by the marketing authorization holder of polaprezinc. Randomized placebo-controlled trials that reported the serum zinc concentration were eligible. The mean difference of the change from baseline in serum zinc concentration was estimated using a fixed-effects model. The linear dose–response relationship and the subgroup effects were also assessed. Out of 54 unique randomized clinical trials (RCTs), four studies met the eligibility criteria, and we could access IPD for all of them. All three doses of polaprezinc (75 mg, 150 mg, and 300 mg) and the placebo group were examined. The dose-combined overall polaprezinc increased the change from baseline by a mean of 9.08 µg/dL (95% confidence interval: 5.46, 12.70; heterogeneity: $I^{2}$ = 0.61%) compared to the placebo. A significant dose–response relationship was confirmed (*p* < 0.001). Baseline serum zinc concentration was considered an effect modifier in polaprezinc 300 mg. All doses of polaprezinc were tolerable, but a dose–response relationship with adverse events (AEs) was observed in gastrointestinal disorders. The dose of 300 mg may be useful among patients with baseline serum zinc concentration of less than 70 µg/dL, and 150 mg for 70 µg/dL or more.

## 1. Introduction

Zinc is an essential trace element that plays a key role in many physiological processes in humans. It also serves as the active component of approximately 300 enzymes [1]. Zinc deficiency is defined as having insufficient zinc in the body to maintain physiological functions. Zinc deficiency may lead to a variety of symptoms and diseases, such as type 1 diabetes [2], autoimmune diseases [3,4,5], growth retardation [6], taste disturbances [7], and skin disorders [8]. In addition, anorexia, hepatitis C, cirrhosis, renal failure, sexual dysfunction, and so on have been reported as diseases or conditions associated with zinc deficiency. The prevalence of zinc deficiency has been estimated at 7.5% to 30% around the world [9].

If zinc deficiency is suspected, or if there are obvious symptoms of zinc deficiency, supplementation with zinc is recommended [10,11]. Polaprezinc is an oral chelate compound consisting of zinc and L-carnosine, which contains 34 mg/150 mg zinc. The protective effect of polaprezinc at 150 mg/day on gastric mucosa has been shown, and it has been approved for treatment of gastric ulcers in Japan and Korea [12]. Clinical studies have also suggested its efficacy on pathological conditions related to zinc deficiency, such as oral mucositis, esophagitis, proctitis, and dermatitis, during and after radiotherapy [13]. It has been used for zinc replacement therapy in clinical practice [14], but is not approved for treatment of zinc deficiency worldwide. Polaprezinc is expected to be effective, but the appropriate regimen of polaprezinc for patients with zinc deficiency is unknown.

In this study, we conducted a systematic review of randomized clinical trials (RCTs) to confirm the potential efficacy and safety of polaprezinc for treatment of zinc deficiency. In addition, we evaluated the dose–response relationships and explored the predictive factors to provide additional information on the appropriate regimen in replacement therapy for zinc deficiency by using individual patient data (IPD).

## 2. Materials and Methods

We registered the study protocol (Supplementary Document 1) with the International Prospective Register of Systematic Reviews (PROSPERO), with the registration number CRD42020156015, and complied with the Preferred Reporting Items for Individual Patient Data systematic reviews (PRISMA-IPD) guidelines [15] in reporting our work.

### 2.1. Search Strategy

We searched PubMed (1946 to October 2019) and the Japanese database Ichushi (1959 to October 2019). The search strategy included only terms describing the intervention and the trial design. In PubMed, the following search strategy was used: (“polaprezinc” [All Fields]) AND clinical trial [ptyp]). An equivalent search strategy was used in the Japanese database Ichushi. No restrictions on language were imposed. To avoid publication bias, the database owned by the marketing authorization holder of polaprezinc (Promac^®^)—Zeria pharmaceutical Co., Ltd. (2512-1, Oshikiri, Konan-machi, Ohsato-gun, Saitama, Japan)—was also accessed. The search strategy used for the database owned by the marketing authorization holder included studies pre-registered with any public clinical trial registration agencies (e.g., the Japan Pharmaceutical Information Center (JAPIC)).

### 2.2. Inclusion and Exclusion Criteria

All studies were included if they fulfilled the following criteria: (1) RCTs; (2) included patients with hypozincemia; (3) compared a single-agent polaprezinc versus a single-agent placebo; and (4) collected the serum zinc concentration. The exclusion criterion was as follows: (1) treatment period of less than 8 weeks. 

### 2.3. Risk-of-Bias Assessment 

We used Version 2 of the Cochrane risk-of-bias tool for randomized trials (RoB2) [16] for risk-of-bias assessment. All included studies were evaluated according to the following domains: bias arising from the randomization process, bias due to deviations from intended interventions, bias due to missing outcome data, bias in measurement of the outcome, bias in selection of the reported result and overall bias. No exclusions were made based on quality.

### 2.4. Data Extraction

We were able to access the IPD of all included studies, although we originally assumed a hybrid meta-analysis of aggregate data and IPD. Details of the study identification are shown in Section 3.1. 

We extracted individual patient-level variables. The extracted explanatory variables included allocated treatment, demographic characteristics (sex, age, presence/absence of comorbidities, baseline serum zinc concentration), and adherence to treatment. The extracted outcomes included serum zinc concentrations, AEs, and adverse drug reactions (ADRs). The uniformed data acceptable time window for each visit was applied to the serum zinc concentrations across studies. To uniform definitions across studies, AEs/ADRs were re-coded using the Medical Dictionary for Regulatory Activities (MedDRA) term system (Version 21.1).

### 2.5. Statistical Analysis

The primary efficacy outcome was the change from baseline in serum zinc concentration at 8 weeks. Secondary efficacy outcomes were the normal proportion defined by the serum zinc concentration of 80 μg/dL or more at 8 weeks and the response proportion defined by the change of serum zinc concentration of 15 μg/dL or more at 8 weeks. Safety outcomes were the incidence of AEs/ADRs and serum concentrations of copper and iron. The diagnosis of zinc deficiency is usually made upon a serum zinc concentration < 70 µg/dL [10]. However, definitions with thresholds of < 80 µg/dL have also been reported [17,18]. Thus, we performed analysis for the following two analysis populations: (1) patients with baseline serum zinc concentration of less than 70 µg/dL (primary analysis population) and (2) patients with baseline serum zinc concentration of less than 80 µg/dL (secondary analysis population). All efficacy analyses were performed for the dose-combined overall polaprezinc and for each dose of polaprezinc (75 mg, 150 mg, and 300 mg). 

For each efficacy outcome, we performed a one-stage fixed-effect IPD meta-analysis to obtain treatment effects with 95% confidence intervals (CIs) and subgroup effects. For continuous outcomes, the mean difference was estimated using linear models, adjusting study and baseline serum zinc concentration as fixed-effect covariates. For binomial outcomes, the risk difference was estimated with linear probability models by the modified least-squares method, adjusting study as a fixed-effect covariate [19,20]. Heterogeneity of treatment effects between trials was tested with the interaction term between treatment and study as a fixed effect. The magnitude of heterogeneity was assessed using the $I^{2}$ statistic, estimated by the interaction term between study and treatment as a random effect.

To evaluate the dose–response relationship, the contrast for linearity was tested with a one-stage fixed effect model, including all treatment groups simultaneously. To evaluate pre-specified intervention effect modifiers (sex, age, comorbidity, and baseline serum zinc concentration), the covariate–treatment interaction terms were included and tested separately at alpha level 20%. In addition, treatment effects within each subgroup were analyzed.

For safety outcomes, incidence proportions of AEs and ADRs were calculated. In addition, serum concentrations of copper and iron were summarized. 

All analyses were pre-specified in a statistical analysis plan finalized prior to conducting the meta-analysis (Supplementary Document 2). The software SAS 9.4 was used for all statistical analyses except for the description of the forest plot, for which R was used.

## 3. Results

### 3.1. Study Characteristics

**Figure 1:**
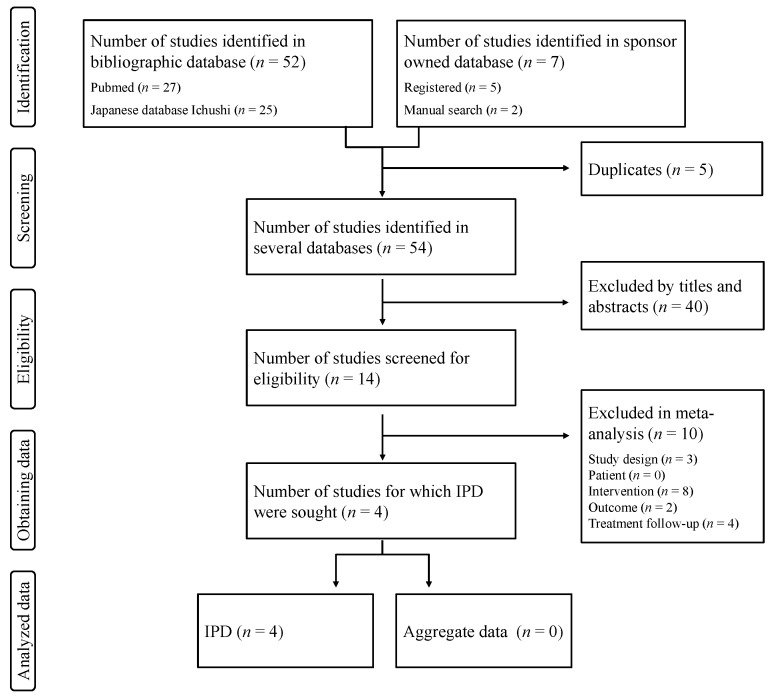
Preferred Reporting Items for Individual Patient Data (PRISMA) flow diagram for this individual patient data (IPD) systematic review.

The search identified 27, 25, and 7 studies in PubMed, the Japanese database Ichushi, and the sponsor-owned database, respectively. After removing duplicates, there were 54 unique studies. Of these, 40 studies were excluded based on screening of the title and abstract. Full texts of 14 studies were assessed to determine inclusion and exclusion, and 10 studies were excluded. The major reason for exclusion was that the comparator was not a placebo. Ultimately, 4 studies met the eligibility criteria, and all of them had accessible IPD, as seen in Figure 1. Two of them were published in 2009 and 2013 [21,22]; the other two studies were unpublished, and were found in the sponsor-owned database [23,24]. All studies were conducted in Japan by Zeria Pharmaceutical Co., Ltd. to evaluate efficacy on taste-disorder-related zinc deficiency. The mean age of subjects was between 43.3 and 45.3 years; the mean baseline zinc concentration was between 71.0 and 77.8 μg/dL. The length of the treatment period was 12 weeks in all studies. There was a slight difference in the interventions across studies. All studies included a polaprezinc 150 mg group. A polaprezinc 300 mg group was included in two studies, and a polaprezinc 75 mg group was included in one study, as shown in Table 1.

From the included studies, we extracted the patients with zinc deficiency as defined above. The number of patients for primary and secondary analysis populations are presented in Table 2. 

The risk-of-bias assessment conducted on the included studies is presented in Figure S1. The following sources were used to help inform the risk-of-bias assessment: journal article, study protocol, statistical analysis plan, clinical study report, and study registry records in the database owned by the marketing authorization holder. All studies were done under good clinical practice (GCP) conditions and had a low risk of bias in all domains. 

### 3.2. Serum Zinc Concentration

**Figure 2:**
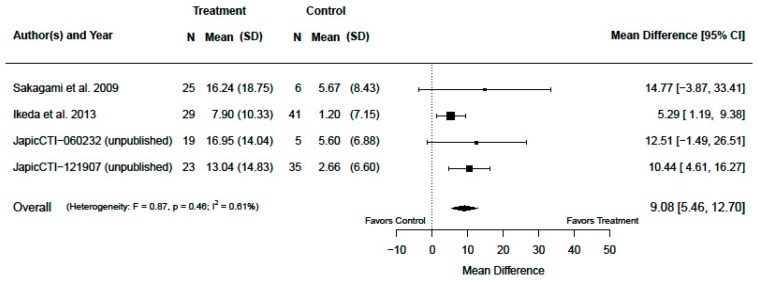
Change from baseline in serum zinc concentrations (µg/dL) (dose-combined overall polaprezinc vs. placebo for the primary analysis population, patients with serum zinc concentrations of less than 70 µg/dL).

**Figure 3:**
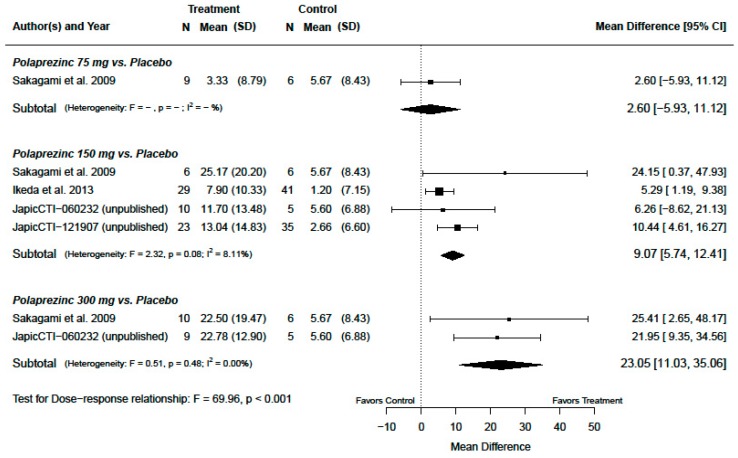
Change from baseline in serum zinc concentrations (µg/dL) (by dose of polaprezinc vs. placebo for the primary analysis population, patients with serum zinc concentrations of less than 70 µg/dL).

The treatment effects of change from baseline in serum zinc concentration for primary analysis population are presented in Figure 2 and Figure 3. The dose-combined overall polaprezinc increased the change from baseline in serum zinc concentration by a mean of 9.08 µg/dL (95% CI: 5.46, 12.70; *p* < 0.001) with $I^{2}$ = 0.61% (*p* = 0.46), as seen in Figure 2. A significant dose–response relationship was confirmed (*p* < 0.001), as seen in Figure 3. The mean differences were 2.60 µg/dL (95% CI: −5.93, 11.12; *p* = 0.52), 9.07 µg/dL (95% CI: 5.74, 12.41; *p* < 0.001), and 23.05 µg/dL (95% CI: 11.03, 35.06; *p* = 0.001) for polaprezinc 75 mg, 150 mg, and 300 mg, respectively. 

The treatment effects of the normal proportion of the primary analysis population are presented in Figures S2 and S3. The dose-combined overall polaprezinc increased the normal proportion, defined as serum zinc concentration over 80 μg/dL, by 19.5% (95% CI: 10.0, 29.0; *p* < 0.001) with $I^{2}$ = 3.32% (*p* = 0.04), as seen in Figure S2. For polaprezinc 75 mg/day, the risk difference was not estimable because there was no patient judged as “normal” in both groups. For 150 mg and 300 mg, the risk differences were 16.4% (95% CI: 6.4, 26.5; *p* = 0.002) and 63.1% (95% CI: 38.2, 87.9; *p* < 0.001), respectively, as seen in Figure S3. The dose–response relationship was confirmed (*p* < 0.001). For response proportion, similar results were obtained, as seen in Figures S4 and S5. 

For the secondary analysis population, the findings were generally similar to the primary population (Figures S6–S11). The only exception was that the treatment effects on binomial outcomes were estimable for polaprezinc 75 mg.

### 3.3. Interaction Effect and Subgroup Analysis

We assessed the differential effects of treatments in subgroups defined by each covariate factor. The covariate–treatment interactions of change from baseline were evaluated using multivariate models, as shown in Table S1. For polaprezinc at 300 mg/day, there was a −21.82 µg/dL (95% CI: −50.42, 6.78; *p* = 0.13) decrease in the treatment effect for every 10.0 µg/dL increase in baseline zinc concentration for the primary analysis population. Similarly, a −11.42 µg/dL (95% CI: −19.62, −3.22; *p* = 0.007) decrease was noted for the secondary analysis population. For the other doses of polaprezinc (overall polaprezinc, 75 mg/day and 150 mg/day), a covariate–treatment interaction was not clear. 

**Figure 4:**
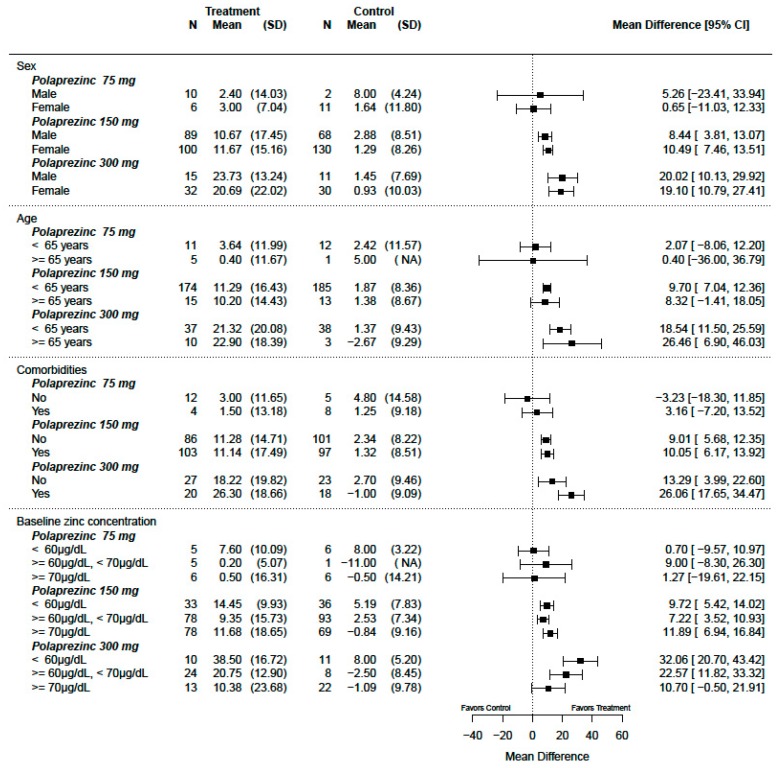
Subgroup analysis for changes from baseline in serum zinc concentrations (µg/dL) (by dose of polaprezinc vs. placebo for the secondary analysis population, patients with serum zinc concentrations of less than 80 µg/dL).

The treatment effects within each covariate factor among broadly defined deficient patients (the secondary analysis population) are presented in Figure 4. There was effect modification for baseline zinc concentration in a comparison between polaprezinc 300 mg and placebo. For the other covariates (sex, age, and comorbidities), effect modifications were not clear, as shown in Table S1 and Figure S12. For the other efficacy outcomes, similar results were obtained. 

### 3.4. Safety

The safety profile was similar in the primary and secondary analysis populations. As the secondary analysis population was about three times larger than the primary analysis population, the results for that population are presented herein. 

The incidence proportions of ADRs were as follows: 14.6% (37/253) for overall polaprezinc, 12.5% (2/16) for polaprezinc 75 mg, 11.1% (21/190) for polaprezinc 150 mg, 29.8% (14/47) for polaprezinc 300 mg, and 14.6% (29/198) for placebo. ADRs related to gastrointestinal disorders were the most frequently observed and implied a dose–response relationship: 0.0% (0/16) for polaprezinc 75 mg, 2.1% (4/190) for polaprezinc 150 mg, 14.9% (7/47) for polaprezinc 300 mg, and 3.0% (6/198) for placebo. ADRs leading to discontinuation of allocated treatment included abdominal discomfort (one patient in the polaprezinc 300 mg group [23]), abdominal distension (one patient in the placebo group [23]), and eczema (one patient in the polaprezinc 150 mg group [22]). No serious ADR was confirmed. All ADRs observed for primary and secondary analysis populations are listed in Tables S2 and S3. 

We assessed serum copper and iron concentrations for up to 12 weeks. There was a slight difference between the placebo and polaprezinc groups, but no sign of copper or iron deficiency was found over the 12 weeks, as shown in Table S4. 

## 4. Discussion

This prospectively planned IPD systematic review provided evidence that polaprezinc increases serum zinc concentration significantly in patients with zinc deficiency, and that there is a significant dose–response relationship. There are several forms of supplemental zinc on the market, such as zinc acetate, zinc gluconate, and zinc sulfate. However, whether differences exist among these forms of zinc in terms of absorption, bioavailability, or tolerability is unclear. It is well known that there is an association between zinc intake and serum zinc concentration [25,26]; however, there is insufficient evidence to define the proper use of certain zinc forms. This review is unique in providing information on the proper use of polaprezinc using IPD meta-analysis. 

We pooled four RCTs and confirmed that dose-combined overall polaprezinc resulted in a significant increase from baseline in serum zinc concentration. Studies included in this review were similar in design, target population, interventions, and treatment period due to our strict eligibility criteria. Therefore, as expected, the heterogeneity in treatment effects was very small ($I^{2}$ = 0.61%; *p* = 0.46), as seen in Figure 2. In an earlier systematic review, which evaluated the relationship between zinc intake and serum/plasma zinc concentration in children, a large heterogeneity was observed between the studies ($I^{2}$ = 97.6%; *p* = 0.0001) [26]. The authors of that review considered that the heterogeneity was due to study population characteristics, doses and dosages of zinc, study duration, and a lack of standardization of dietary/laboratory assessment methods. We attempted to accurately describe the biological relationship between polaprezinc dose and serum zinc concentration under strict experimental conditions; therefore, this small heterogeneity was desirable for our purpose.

A dose–response relationship was confirmed for all efficacy outcomes. There was a 23.05 µg/dL (95% CI: 11.03, 35.06) increase in the treatment effect of the serum zinc concentration upon polaprezinc 300 mg administration in the primary analysis population. This effect size was reasonable compared to existing approved zinc preparations for zinc deficiency. Zinc acetate 50 mg, which contains 50 mg of zinc, had a treatment effect of a 22.4 µg/dL increase in a randomized placebo-controlled trial with the same parameters (same eligibility criteria for severity of zinc deficiency, duration of treatment, comparator, and endpoint) [27]. Zinc acetate 50 mg has proven efficacy in the treatment of Wilson’s disease and hypozincemia. This implies that polaprezinc 300 mg may be potentially effective in these conditions as well.

This review showed an association between baseline serum zinc concentrations and treatment effects with 300 mg polaprezinc. The effect size of 300 mg polaprezinc in the change from baseline among the patients with baseline zinc concentrations higher than 70 µg/dL was smaller than other effects, and was comparable to that of 150 mg polaprezinc, as seen in Figure 4. It is known that free zinc concentrations in the cell are regulated by some zinc transporters and metallothioneins (small metal-binding cysteine-rich proteins with a high affinity for zinc) [28,29]. Zinc concentration elevation in the cell induces synthesis of metallothioneins, and the synthesized metallothioneins bind the zinc in the cell until the zinc concentration drops to a certain point [28,29]. It has been reported that the amount of metallothionein-bound zinc increases only when the plasma zinc concentration exceeds a critical value (70.9 µg/dL) [18,30]. Considering this biological rationale for the attenuation of serum zinc increase, even if the dose is increased above 300 mg in patients with serum zinc concentrations greater than 70 µg/dL, an increase in serum zinc concentration proportional to the dose increase cannot be expected due to the metallothionein-bound zinc elevation. Therefore, among patients with serum zinc concentrations higher than 70 µg/dL, the usefulness of polaprezinc 300 mg is debatable and polaprezinc 150 mg may be sufficient. On the other hand, among patients with serum zinc concentrations of less than 70 µg/dL, polaprezinc 300 mg has advantages compared to polaprezinc 150 mg.

In terms of safety, we observed acceptable tolerability of polaprezinc. Zinc toxicity can occur in both acute and chronic forms. Acute adverse effects of zinc intake include epigastric pain, nausea, vomiting, loss of appetite, abdominal cramps, diarrhea, and headaches [11]. Zinc intake is also associated with chronic effects such as low copper status, altered iron function, some functional impairment in immunological response, and decreasing levels of serum lipoprotein and cholesterol concentrations [11]. In the current review, no unexpected safety concerns were observed. Note that the incidence of gastrointestinal disorders was suggested to increase in a dose–response manner. Reductions in copper-containing enzyme, a marker of copper status, have been reported with even moderately high zinc intakes of approximately 60 mg/day for up to 10 weeks [11]. A potential trade-off relationship in dose was suggested for efficacy and safety.

This review had four main limitations. First, patients included in this meta-analysis appeared not to represent zinc-deficient patients in two aspects: disease background and age category (adult/children). All patients included in the current review had taste disorders. It is well known that zinc deficiency has been described in various diseases and varies in its cause [10]. Appropriate doses and clinically meaningful effects may vary among patients with zinc deficiencies with different backgrounds. In addition, the recommended amount of daily zinc intake varies by age [11]. In fact, many of the zinc preparations for zinc deficiency differ in approved doses for adults and children. As the appropriate dose of polaprezinc should vary for adults and children, it is difficult to extrapolate the results of this review to children. Second, the change from baseline in serum zinc concentration may not show sufficient surrogacy for zinc deficiency [31,32]. It has been reported that a low serum zinc concentration is related to clinical signs of zinc deficiency, and can be used as a biomarker of zinc status with progressively lower serum zinc concentrations. However, an increase in zinc concentration does not necessarily reflect an increase in cellular zinc status in high serum zinc concentrations, due to tight homeostatic control mechanisms [31]. In addition, serum zinc concentrations are affected by biological factors such as infection/inflammation, stress and hormones [33]. Various factors and conditions should be considered in interpretation of the association between zinc concentrations and zinc deficiency [31,34]. Third, this review provides no evidence that polaprezinc improves pathological conditions related to zinc deficiency. Further research is warranted regarding the degree to which zinc supplementation is effective in each pathological condition related to zinc deficiency. Finally, all prognostic factors may not have been fully adjusted. Indeed, all included studies were randomized in design; therefore, unmeasured and measured confounders must be balanced within each study. However, it is somewhat possible to compromise within-trial randomization in the process of extracting patients, especially in a small study. 

On the other hand, our review had several strengths. We performed data synthesis using IPD, which contributed to increasing internal validity and reliability. The target population of our review was patients with zinc deficiency. We were able to extract the population of interest by applying uniformed eligibility criteria to patient-level baseline serum zinc concentrations. When analyzing, we applied the models adjusting patient-level baseline serum zinc concentration to an efficacy outcome, the definition of which was standardized across studies. This means that we could estimate the treatment effect accurately by diminishing study-to-study heterogeneity. Furthermore, when evaluating subgroup effects, we were able obtain the pooled effects for specific subgroups and assess patient-level interaction (not study-level interaction derived from meta-regression). Generally, undertaking an IPD meta-analysis requires a lot of time, money, and collaborative work, but it certainly has many advantages. Indeed, sharing the IPD of clinical studies is being promoted. It is hoped that IPD will lead to better evidence of the contribution of zinc intake to alleviating zinc deficiency.

## 5. Conclusions

In conclusion, this IPD systematic review confirmed that polaprezinc significantly increased the serum zinc concentration for patients with zinc deficiency, and that there was a significant dose–response relationship. In addition, we found that the dose–response relationship was attenuated in patients with a baseline serum zinc concentration of 70 µg/dL or higher, and that incidence of gastrointestinal disorders increased dose-dependently. When using polaprezinc as a zinc replacement therapy for zinc deficiency, it may be necessary to determine the appropriate dose in consideration of the patient’s condition.

## Figures and Tables

**Table 1:** Characteristics of the included studies.

Author, Year	Sample Size	Studied Population	Treatments	Male (%)	Age (Means ± SD, Year)	Baseline Zinc (Means ± SD, μg/dL)	Duration of Treatment (Week)
Sakagami et al., 2009 [21]	107	Outpatients with zinc-related taste disorder, aged 20–80 years	Polaprezinc 75 mg/day (*n* = 27) Polaprezinc 150 mg/day (*n* = 25) Polaprezinc 300 mg/day (*n* = 28) Placebo (*n* = 27)	47.4	45.1 ± 16.2	71.0 ± 12.7	12
Ikeda et al., 2013 [22]	219	Outpatients with zinc-related taste disorder, aged 20–75 years	Polaprezinc 150 mg (*n* = 108) Placebo (*n* = 111)	39.7	45.2 ± 12.9	72.7 ± 12.6	12
JapicCTI-060232 * (unpublished) [23]	149	Outpatients with zinc-related taste disorder, aged 20–80 years	Polaprezinc 150 mg (*n* = 47) Polaprezinc 300 mg (*n* = 51) Placebo (*n* = 51)	45.	43.3 ± 13.8	77.8 ± 11.8	12
JapicCTI-121907 * (unpublished) [24]	269	Outpatients with zinc-related taste disorder, aged 20–75 years	Polaprezinc 150 mg (*n* = 134) Placebo (*n* = 135)	50.2	45.3 ± 12.3	76.1 ± 14.1	12

*: the Japan Pharmaceutical Information Center (JAPIC) registration number.

**Table 2:** Number of patents in analysis populations.

Treatments	Original Sample Size	Primary Analysis Population ^†^	Secondary Analysis Population ^‡^
Overall Polaprezinc	420	97	322
Polaprezinc 75 mg	27	9	21
Polaprezinc 150 mg	314	69	241
Polaprezinc 300 mg	324	19	60
Placebo	79	87	255
Total	744	184	577

^†^: patients with serum zinc concentrations of less than 70 µg/dL; ^‡^: patients with serum zinc concentration of less than 80 µg/dL.

## Supplementary Materials

The following are available online at https://www.mdpi.com/2072-6643/12/4/1128/s1, Document S1: Study protocol, Document S2: Statistical analysis plan, Figure S1: Risk-of-bias summary across studies, Figure S2: Normal proportion (%) (dose-combined overall polaprezinc vs. placebo for primary analysis population, Figure S3: Normal proportion (%) (by dose of polaprezinc vs. placebo for primary analysis population, Figure S4: Response proportion (%) (dose-combined overall polaprezinc vs. placebo for primary analysis population, Figure S5: Response proportion (%) (by dose of polaprezinc vs. placebo for primary analysis population, Figure S6: Change form baseline in serum zinc concentration (µg/dL) (dose-combined overall polaprezinc vs. placebo for secondary analysis population, Figure S7: Change form baseline in serum zinc concentration (µg/dL) (by dose of polaprezinc vs. placebo for secondary analysis population, Figure S8: Normal proportion (%) (dose-combined overall polaprezinc vs. placebo for secondary analysis population, Figure S9: Normal proportion (%) (by dose of polaprezinc vs. placebo for secondary analysis population, Figure S10: Response proportion (%) (dose-combined overall polaprezinc vs. placebo for secondary analysis population, Figure S11: Response proportion (%) (by dose of polaprezinc vs. placebo for secondary analysis population, Figure S12: Subgroup analysis for changes from baseline in serum zinc concentration (µg/dL) for primary analysis population, Table S1: Interaction effects of treatment by each covariate factor, Table S2: Adverse drug reactions for primary analysis population, Table S3: Adverse drug reactions for secondary analysis population, Table S4: Serum concentration of copper and iron for secondary analysis population.
