# Supplementary material for: Efficacy and Safety of Polaprezinc (Zinc Compound) on Zinc Deficiency: A Systematic Review and Dose–Response Meta-Analysis of Randomized Clinical Trials Using Individual Patient Data
Source: Nutrients. 2020 Apr 17;12(4):1128. doi: 10.3390/nu12041128 (PMC7230469; doi:10.3390/nu12041128)
Supplement: Supplementary file 1 [file nutrients-12-01128-s001.zip › Supplementary File 1/Document S1_Study Protocol.pdf]

Systematic review of randomized clinical trials to assess the efficacy and safety of polaprezinc for the patients with hypozincemia

Protocol

Version: 1.0

2019/Oct/15

Document History

Ver.	Date	Rationale	Summary of Change
1.0	2019/Oct/15	Create New<br>Agreed by review team members after study kick off meeting on 2019/Oct/15	NA

目次

Introduction 1
Aim of the project 1
Organizational affiliation of the project 1
Review team members and their organizational affiliations 1
Contact details for further information 2
Targeted trials 2
Inclusion criteria 2
Exclusion criteria 2
Search strategy 2
Risk of bias assessment 3
Endpoints 3
Efficacy endpoints 3
Safety endpoints 3
Statistical analysis 3
Publication 4
Reference 4

Introduction

Zinc is a representative essential trace element and its lack causes various diseases, taste disorder, dermatitis, hair loss, anemia, stomatitis, male sexual dysfunction, compromised, osteoporosis and so on. [1]

Polaprezinc (Promac®) has been available as zinc compounds in several countries. This has a protective effect on the gastric mucosa and is indicated for gastric ulcer. However, it has been reported that it is used not only for gastric ulcers but also for zinc deficient patients as zinc replacement therapy.

In this study, we conduct a systematic review of the clinical trials to assess the efficacy and safety of polaprezinc for the patients with hypozincemia based on the serum zinc concentration.

Aim of the project

- To evaluate that polaprezinc is superior to placebo in increasing the serum zinc concentration for the patients with hypozincemia
- To evaluate the dose-response relationship and the magnitude of the effect of each dose
- To explore the prognostic and predictive factors
- To evaluate the safety of polaprezinc

Organizational affiliation of the project

ZERIA Pharmaceutical Co., Ltd.
10-11, Nihonbashi Kobuna-cho, Chuo-ku, Tokyo
103-8351 Japan
TEL: +81-3-3663-0533
FAX: +81-3-3663-0534

Review team members and their organizational affiliations

Masafumi Sakagami, Hyogo College of Medicine, Hyogo, Japan
Takaki Miwa, Kanazawa Medical University, Ishikawa, Japan.
Yuji Naito, Kyoto Prefectural University of Medicine, Kyoto, Japan.
Koji Oba, The University of Tokyo, Tokyo, Japan.
Masaru Tsuchikawa, ZERIA Pharmaceutical Co., Ltd., Tokyo, Japan.

Kei Furihata, ZERIA Pharmaceutical Co., Ltd., Tokyo, Japan.

Contact details for further information

Masaru Tsuchikawa
masaru-tsuchikawa@ZERIA.co.jp

Targeted trials

Inclusion criteria

1. Randomized clinical trials
2. Clinical trials including the patients with hypozincemia
3. Comparing a single-agent polaprezinc versus a single-agent Placebo
4. Clinical trials collecting the serum zinc concentration

Exclusion criteria

1. Clinical trials with treatment period of less than 8 weeks

Search strategy

Pubmed and Japanese Database (Ichu-shi) search will be performed to identify all randomized clinical trials that compared a single-agent polaprezinc to a single-agent placebo. Search strings are the following. No restrictions on language or publication date will be imposed. The formal search will be performed after PROSPERO registration.

Pubmed:
("polaprezinc"[All Fields]) AND (clinical trial [ptyp])

Japanese Database (Ich-shi):
(Polaprezinc/TH OR polaprezinc/AL) AND (PT=原著論文 AND RD=ランダム化比較試験)

To avoid publication bias, database of ZERIA pharmaceutical Co., Ltd., a marketing authorization holder of polaprezinc, will be assessed. Individual participant data will be utilized.

Risk of bias assessment

To assess the methodological quality of the included trials, the criteria for quality assessment recommended in the Cochrane Collaboration Handbook will be used. No exclusions are planned based on quality.

Endpoints

Efficacy endpoints

- the serum zinc concentration

Note: Definition and data handling will be standardized across the component trials as far as possible.

Safety endpoints

- the incidence of adverse events and adverse drug reaction

Note: Each adverse event will be re-coded by the same terminology, MedDRA ver. 21.1, as far as possible.

Statistical analysis

Following integrated analysis will be performed

- Demographics and baseline characteristics
  - demographics and baseline laboratory results will be summarized
- Efficacy analysis
  - Apply the quantitative meta-analysis, where mean difference will be extracted or calculated form available data. Meta-analysis will be carried out using fixed-effect model. The heterogeneity across the component trials will be assessed by I-square statistics calculated by mixed models.
  - Subgroup analyses will be performed following items.
    - age
    - sex
    - the baseline serum zinc concentration
- Safety analysis
  - Tabulate the incidence of adverse events and adverse drug reaction.

Publication

Manuscripts summarizing the meta-analysis work and presenting the results will be prepared for submission to and publication by a peer-reviewed scientific journal.

Reference

1. Kodama, Itakura, Omori et al., "Aenketsubosho no shinryoshishin 2018" [Clinical guideline for zinc deficiency 2018], J. Jpn. Soc. Clin. Nutr., **40**(2):120-167, 2018. (in Japanese)

Accept Protocol – Signature Page

Protocol Name: Systematic review of randomized clinical trials to assess the efficacy and safety of polaprezinc for the patients with hypozincemia Ver. 1.0

Masafumi Sakagami

(Signature) — (Date) Oct. 15, 2019

Takaki Miwa

(Signature) — (Date) Oct. 15, 2019

Yuji Naito

(Signature) — (Date) Oct. 15, 2019

Koji Oba

(Signature) — (Date) Oct 15. 2019.

Masaru Tsuchikawa

(Signature) — (Date) Oct 15. 2019

Kei Furihata

(Signature) — (Date) Oct. 15. 2019
