# Supplementary material for: Efficacy and Safety of Polaprezinc (Zinc Compound) on Zinc Deficiency: A Systematic Review and Dose–Response Meta-Analysis of Randomized Clinical Trials Using Individual Patient Data
Source: Nutrients. 2020 Apr 17;12(4):1128. doi: 10.3390/nu12041128 (PMC7230469; doi:10.3390/nu12041128)
Supplement: Supplementary file 1 [file nutrients-12-01128-s001.zip › Supplementary File 1/Document S2_Statistical Analysis Plan.pdf]

Systematic review of randomized clinical trials to assess the efficacy and safety of polaprezinc for the patients with hypozincemia

Statistical Analsis Plan

Version: 1.0

5/Nov/2019

Document History

Ver.	Date	Rationale	Summary of Change
1.0	5/Nov/2019	Create New	NA

Index

1. Aim of the present document 1
2. Analysis population 1
3. Treatment comparison 1
4. Endpoints 2
4.1. Efficacy endpoints 2
4.2. Safety endpoints 2
5. Statistical methods 2
5.1.1. Efficacy endpoints 2
5.1.1.1. Continuous endpoints 2
5.1.1.2. Binary endpoints 3
5.1.2. Safety endpoints 4
Reference 4
Accept Statistical analysis plan – Signature Page 5
Appendix 1. the acceptable window of the data for each efficacy endpoint 6

1. Aim of the present document

The purpose of the present document is to provide details on the statistical methods that will be used in the meta-analysis. This document is based on the protocol (dated 15/Oct/2019).

2. Analysis population

The following analysis set will be defined. Both analysis set will be used for all efficacy and safety analysis.

- Primary analysis set

  The primary analysis set is defined as all randomized patients excluding the following patients.

  - Patients who are GCP non-compliant
  - Patients who failed to take the assigned treatment
  - Patients whose serum zinc concentration before randomization exceed 70μg/dL
  - Patients who need to be excluded from a medical point of view

- Exploratory analysis set

  The exploratory analysis set is defined as all randomized patients excluding the following patients.

  - Patients who are GCP non-compliant
  - Patients who failed to take the assigned treatment
  - Patients whose serum zinc concentration just before randomization exceed 80μg/dL
  - Patients who need to be reviewed from a medical point of view

3. Treatment comparison

The following treatment comparisons will be evaluated.

- Polaprezinc (any dose) versus placebo
- Polaprezinc 75mg/day versus placebo
- Polaprezinc 150mg/day versus placebo
- Polaprezinc 300mg/day versus placebo

4. Endpoints

4.1. Efficacy endpoints

- the change of serum zinc concentration from baseline to week 8
- the response based on the serum zinc concentration judged by threshold of 80μg/dL or more at week 8
  - Response at week 8 is defined whether or not the final value of the zinc concentration up to week 8 is equal to or larger than 80μg/dL. If subjects discontinue by that week, he/she is judged as non-responder.
- the response based on the change of serum zinc concentration from baseline judged by threshold of 15μg/dL or more at week 8
  - Response at week 8 is defined whether or not the final value of the change from baseline for zinc concentration up to week 8 is equal to or larger than 15μg/dL. If subjects discontinue by that week, he/she is judged as non-responder.
- Note: The above efficacy endpoints will also be evaluated at week 4 and 12.

* refer appendix 1 for details on acceptable data

4.2. Safety endpoints

- the incidence of adverse events and adverse drug reactions
- the serum iron concentration
- the serum copper concentration
- the serum zinc concentration

5. Statistical methods

The following analysis will be performed by one stage method using individual patient data.

5.1.1. Efficacy endpoints

5.1.1.1. Continuous endpoints

- To estimate the pooled treatment effect across trials
  - A linear fixed-effect model, with trial and baseline serum zinc concentration as fixed-effects, will be applied.
  - Heterogeneity of treatment effects between trials will be evaluated by a fixed interaction term between treatment and trial. The magnitude of heterogeneity

will be assessed by I-square statistics which is derived by a multi-level (patients nested in trials) mixed-effects model.

  - Forest plot, including treatment effects for component trials and pooled treatment effect, will be displayed for each treatment comparison.

- To evaluate the dose-response relationship
  - A linear fixed-effect model, with trial and baseline serum zinc concentration as fixed-effects, will be applied. The dose-response relationship will be tested by contrast for linear dose-response relationship.
  - Note that above analysis will be performed using all trials including polaprezinc 75mg/150mg/300mg or placebo group.
- To explore predictive factors
  - Subgroup analysis will be performed for each biomarker (age, sex, baseline serum zinc concentration, and presence/absence of co-morbidity).
  - A multi-variate linear fixed-effects model, with biomarkers and interaction terms between effect and each biomarker (age, sex, baseline serum zinc concentration, and presence/absence of co-morbidity) as fixed-effects, will be applied.
  - In order to avoid the ecological bias, a linear fixed-effects model will be applied for biomarkers with p-value of less than 0.20 in a multi-variate linear model. This model allows modelling of both within-trial and between-trial interaction effect separately [1].

5.1.1.2. Binary endpoints

- To estimate the pooled treatment effect across trials
  - A linear binomial model using the modified least-squares method [2, 3] with trial as a fixed-effect will be applied.
  - Heterogeneity of treatment effects between trials will be evaluated by a fixed interaction term between treatment and trial. The magnitude of heterogeneity will be assessed by I-square statistics which is derived by a multi-level (patients nested in trials) mixed-effects model.
  - Forest plot, including treatment effects for component trials and pooled treatment effect, will be displayed for each treatment comparison.
- To evaluate the dose-response relationship
  - A linear binomial regression model using the modified least-squares method with trial as a fixed-effect will be applied. The dose-response relationship will be tested by contrast for linear dose-response relationship.

- Note that above analysis will be performed using all trials including polaprezinc 75mg/150mg/300mg or placebo group.

- To explore predictive factors
  - Subgroup analysis will be performed for each biomarker (age, sex, baseline serum zinc concentration, and presence/absence of co-morbidity).

5.1.2. Safety endpoints

- the incidence of adverse events and adverse drug reaction
  - The incidence proportion of adverse events and adverse drug reaction will be tabulated.
- the serum iron concentration
  - Serum iron concentration will be summarized.
- the serum copper concentration
  - Serum copper concentration will be summarized.
- the serum zinc concentration
  - Serum zinc concentration will be summarized.

Reference

1. Fisher DJ, et al. A critical review of methods for the assessment of patient-level interactions in individual participant data meta-analysis of randomized trials, and guidance for practitioners. *J Clin Epidemiol.* 2011; 64: 949-967.
2. Cheung YB. A modified least-squares regression approach to the estimation of risk difference. *Am J Epidemiol.* 2007; 166(11):1337-1344.
3. Hagiwara Y, et al. The number of events per confounder for valid estimation of risk difference using modified least-squares regression. *Am J Epidemiol.* 2018; 187(11):2481-2490.

Accept Statistical analysis plan – Signature Page

Protocol: Systematic review of randomized clinical trials to assess the efficacy and safety of polaprezinc for the patients with hypozincemia Ver. 1.0

Koji Oba

Koji Oba | 5 Nov 2019

(Signature) | (Date)

Masaru Tsuchikawa

Masaru Tsuchikawa | 5 Nov 2019

(Signature) | (Date)

Kei Furihata

Kei Furihata | 5 Nov 2019

(Signature) | (Date)

Appendix 1. the acceptable window of the data for each efficacy endpoint

<table>
<tr><th rowspan="2">Efficacy endpoints</th><th colspan="3">Treatment period</th><th>Note</th></tr>
<tr><th>Week 4</th><th>Week 8</th><th>Week 12</th><th></th></tr>
<tr><td>the change from baseline for serum zinc concentration</td><td rowspan="3">Day 2 - Day 43</td><td rowspan="3">Day 2 - Day 71</td><td rowspan="3">Day 2 - Day 99</td><td>Adopt the final serum zinc concentration within the acceptable window (including data at the time of trial discontinuation)</td></tr>
<tr><td>the response based on the serum zinc concentration judged by threshold of 80μg/dL or more</td><td rowspan="2">Adopt the final serum zinc concentration within the acceptable window (excluding data at the time of trial discontinuation since trial discontinuation will be considered as non-response)</td></tr>
<tr><td>the response based on the change of serum zinc concentration from baseline judged by threshold of 15μg/dL or more</td></tr>
</table>

* Day: The date of serum zinc concentration − the randomization date + 1
