# Supplementary material for: Efficacy and Safety of Polaprezinc (Zinc Compound) on Zinc Deficiency: A Systematic Review and Dose–Response Meta-Analysis of Randomized Clinical Trials Using Individual Patient Data
Source: Nutrients. 2020 Apr 17;12(4):1128. doi: 10.3390/nu12041128 (PMC7230469; doi:10.3390/nu12041128)
Supplement: Supplementary file 1 [file nutrients-12-01128-s001.zip › Supplementary File 1/Supplementary Figures and Tables.pdf]

Supplementary Figures and Tables

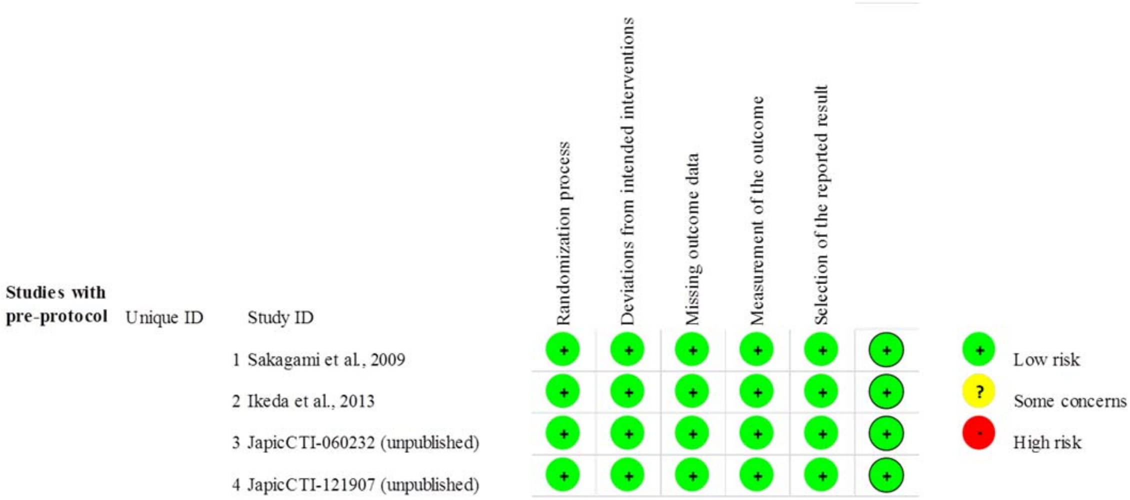

Figure S1. Risk-of-bias summary across studies. Low risk of bias: "+"; Some concerns of bias: "?", "!"; High risk of bias: "-".

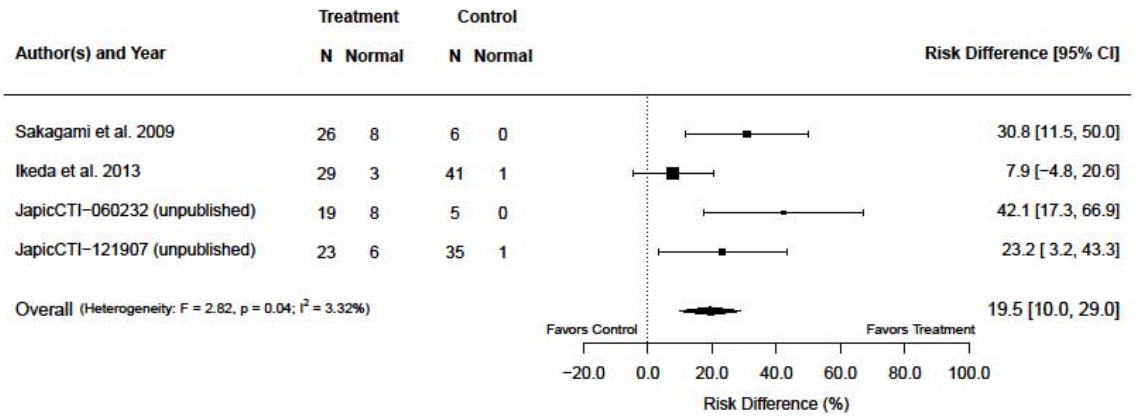

Figure S2. Normal proportion (%), defined by serum zinc concentration of 80 μg/dL or more (dose-combined overall polaprezinc vs. placebo for primary analysis population, patients with serum zinc concentration of less than 70 μg/dL).

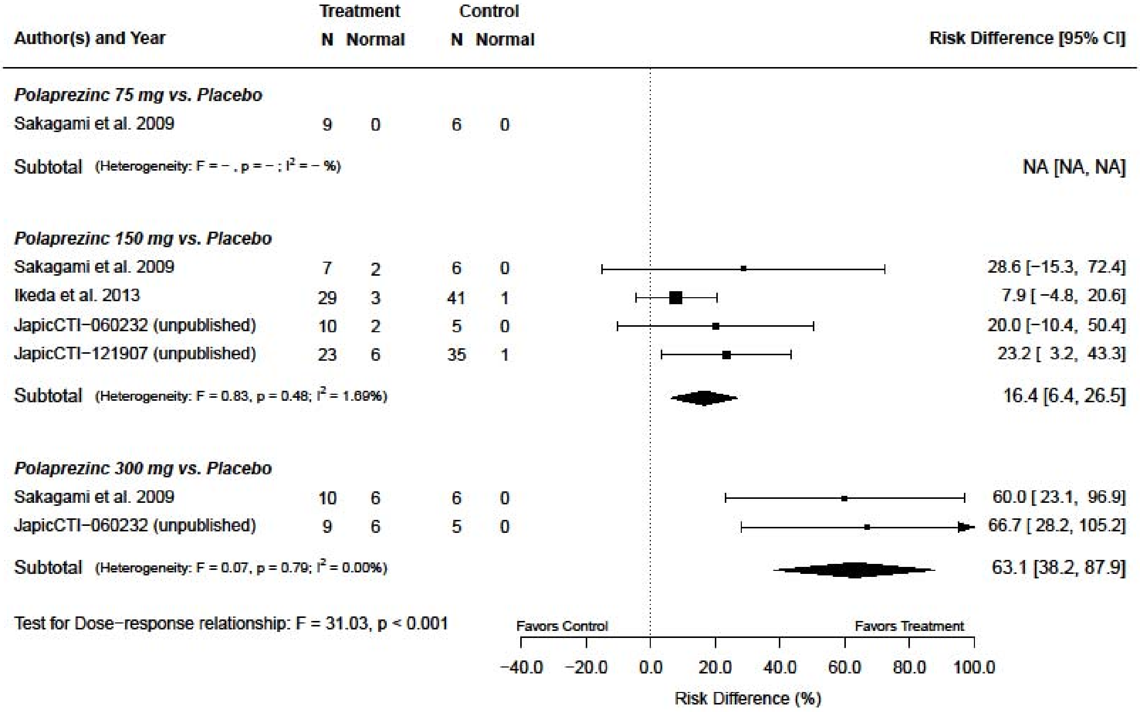

Figure S3. Normal proportion (%), defined by serum zinc concentration of 80 µg/dL or more (by dose of polaprezinc vs. placebo for primary analysis population, patients with serum zinc concentration of less than 70 µg/dL).

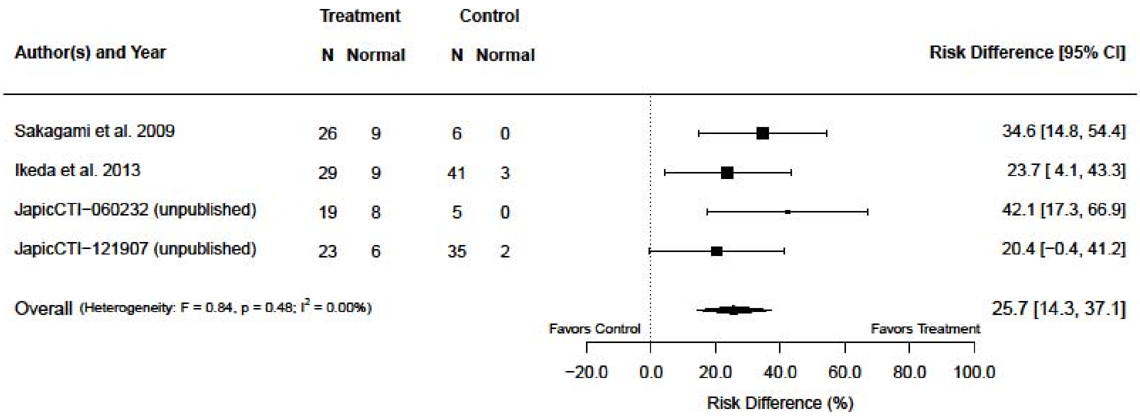

Figure S4. Response proportion (%), defined by change of serum zinc concentration of 15 μg/dL or more (dose-combined overall polaprezinc vs. placebo for primary analysis population, patients with serum zinc concentration of less than 70 μg/dL).

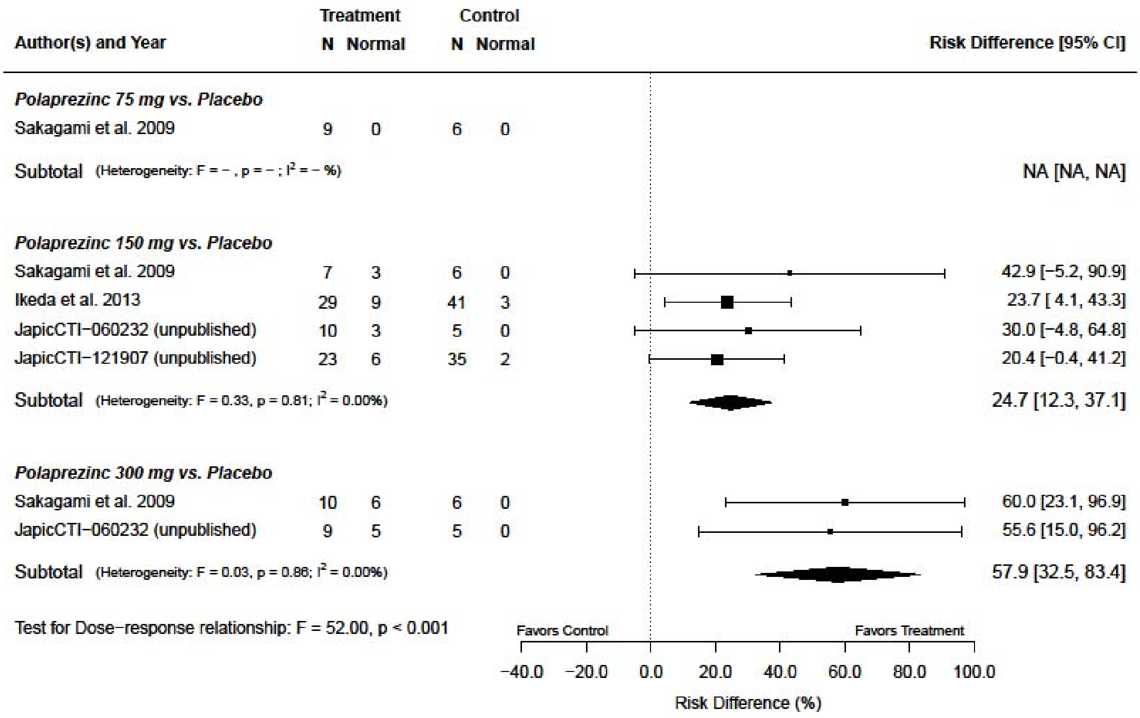

Figure S5. Response proportion (%), defined by serum zinc concentration of 80 μg/dL or more (by dose of polaprezinc vs. placebo for primary analysis population, patients with serum zinc concentration of less than 70 μg/dL).

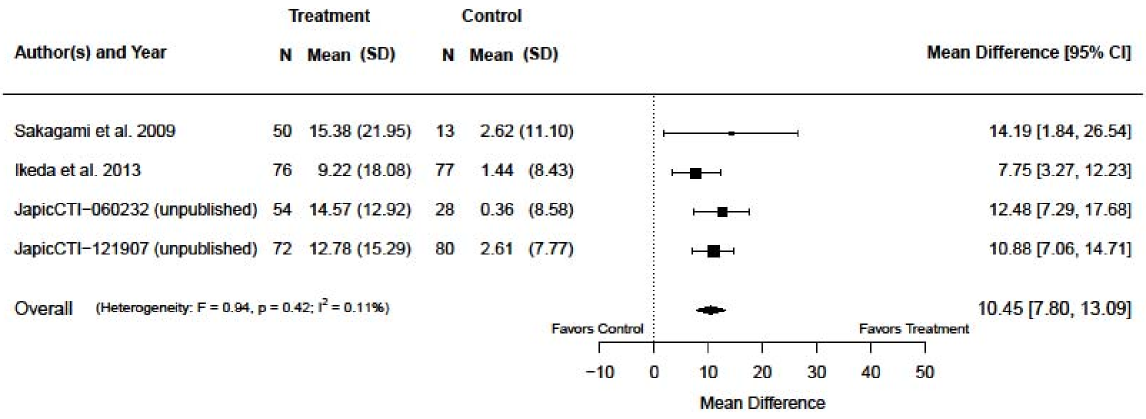

Figure S6. Change form baseline in serum zinc concentration (μg/dL) (dose-combined overall polaprezinc vs. placebo for secondary analysis population, patients with serum zinc concentration of less than 80 μg/dL).

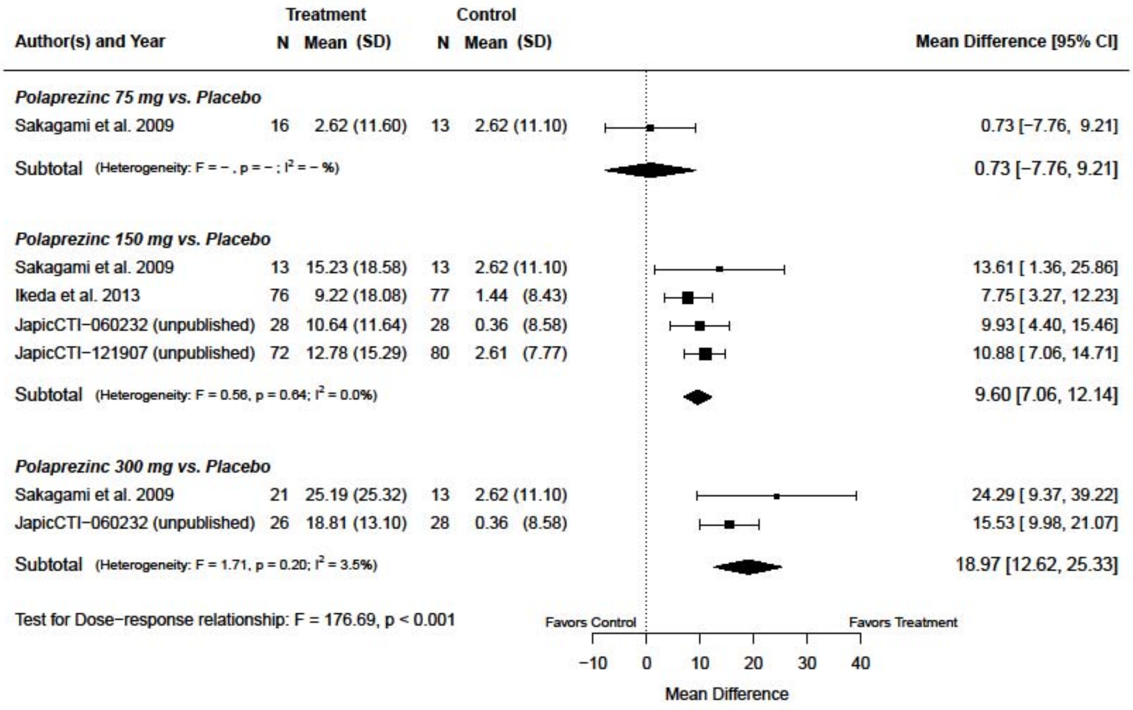

Figure S7. Change form baseline in serum zinc concentration (μg/dL) (by dose of polaprezinc vs. placebo for secondary analysis population, patients with serum zinc concentration of less than 80 μg/dL).

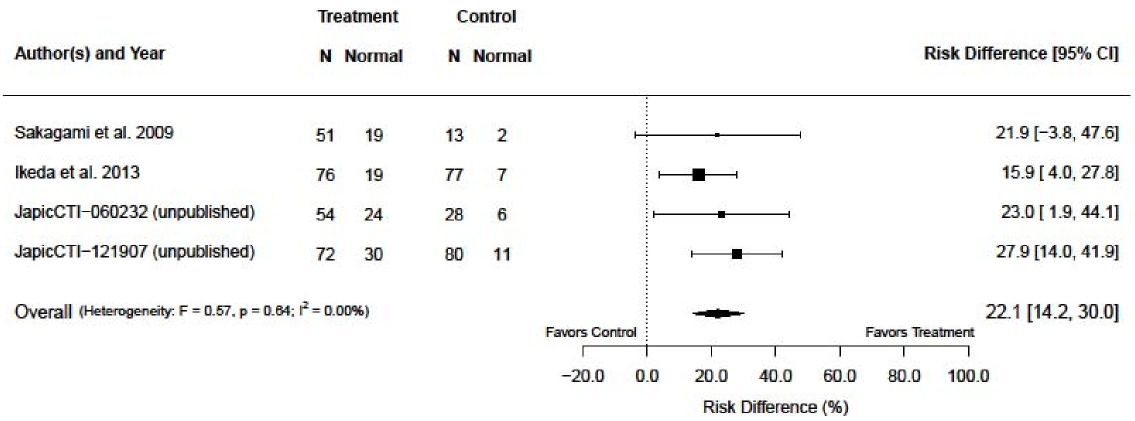

Figure S8. Normal proportion (%), defined by serum zinc concentration of 80 μg/dL or more (dose-combined overall polaprezinc vs. placebo for secondary analysis population, patients with serum zinc concentration of less than 80 μg/dL).

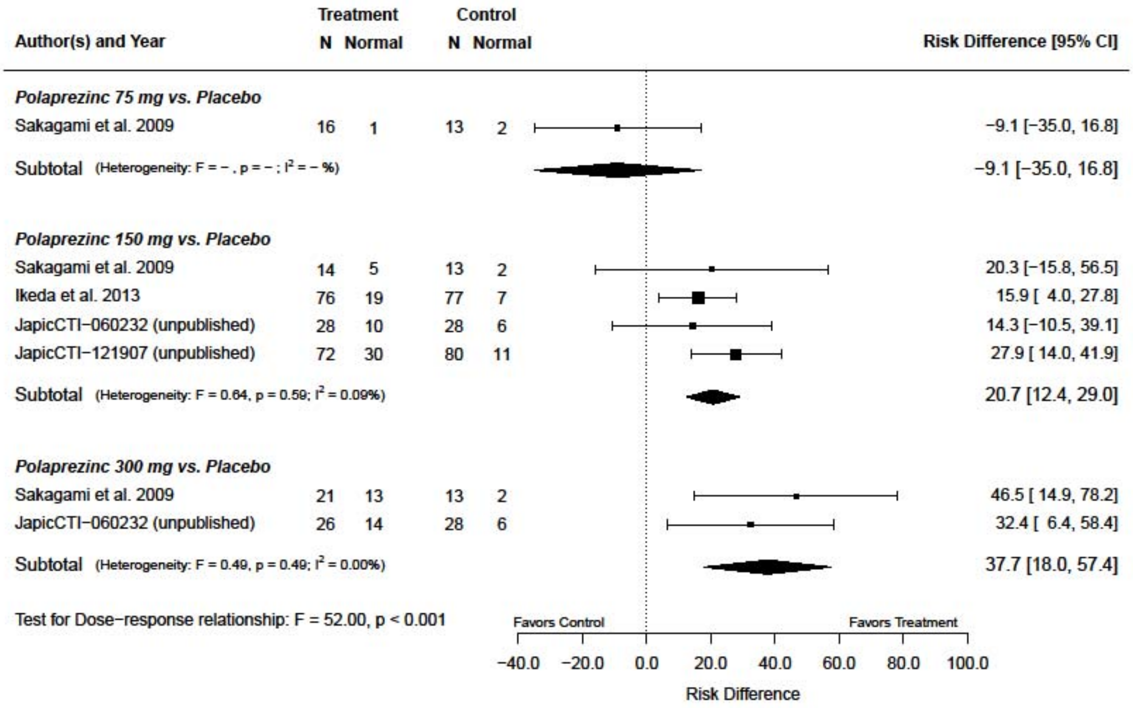

Figure S9. Normal proportion (%), defined by serum zinc concentration of 80 μg/dL or more (by dose of polaprezinc vs. placebo for secondary analysis population, patients with serum zinc concentration of less than 80 μg/dL).

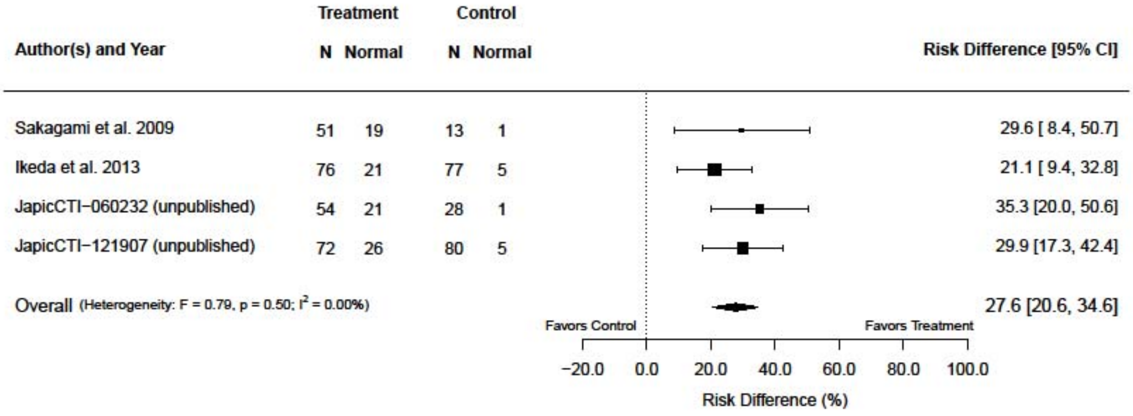

Figure S10. Response proportion (%), defined by change of serum zinc concentration of 15 μg/dL or more (dose-combined overall polaprezinc vs. placebo for secondary analysis population, patients with serum zinc concentration of less than 80 μg/dL).

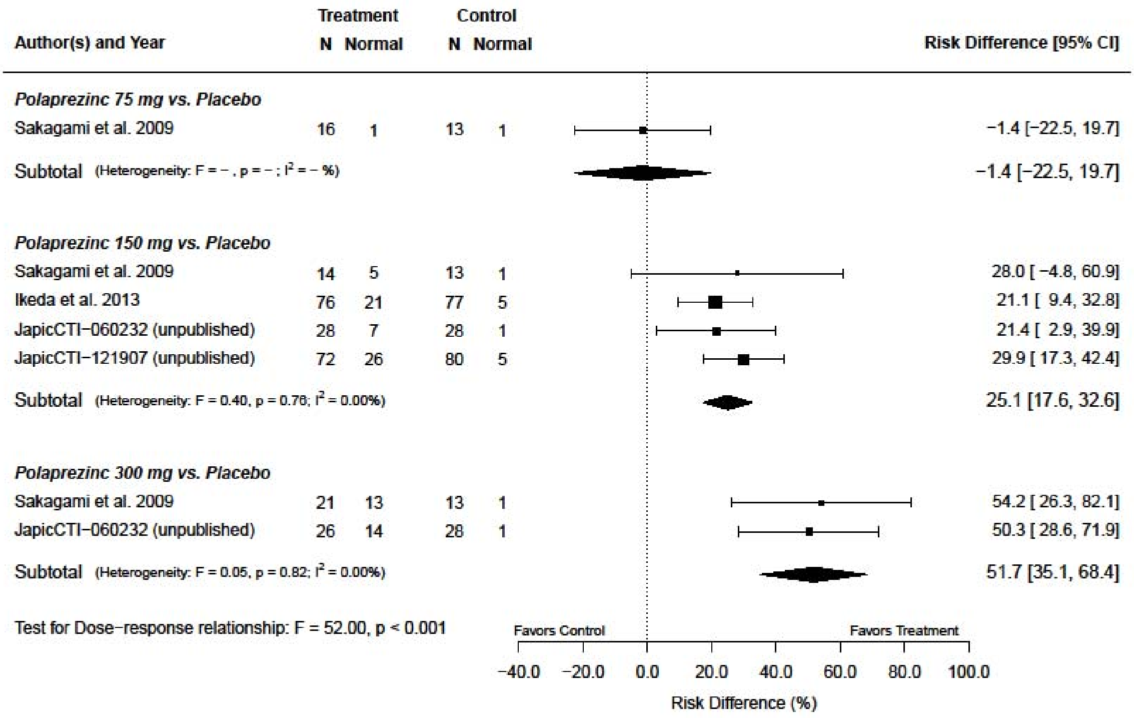

Figure S11. Response proportion (%), defined by change of serum zinc concentration of 15 μg/dL or more (by dose of polaprezinc vs. placebo for secondary analysis population, patients with serum zinc concentration of less than 80 μg/dL).

	Treatment N	Treatment Mean	Treatment (SD)	Control N	Control Mean	Control (SD)	Mean Difference [95% CI]
**Sex**							
Polaprezinc 75 mg							
Male	5	4.00	(10.22)	2	8.00	(4.24)	5.65 [−14.58, 25.87]
Female	4	2.50	(8.06)	4	4.50	(10.34)	0.51 [−14.51, 15.52]
Polaprezinc 150 mg							
Male	28	11.82	(16.28)	21	2.38	(7.70)	9.26 [1.26, 17.26]
Female	40	11.65	(12.29)	66	2.33	(6.86)	8.51 [4.96, 12.05]
Polaprezinc 300 mg							
Male	8	25.38	(14.86)	4	7.00	(5.60)	16.34 [−9.44, 42.12]
Female	11	20.64	(17.59)	7	4.86	(8.55)	24.26 [9.17, 39.34]
**Age**							
Polaprezinc 75 mg							
< 65 years	5	4.40	(5.27)	5	5.80	(9.42)	3.03 [−5.91, 11.96]
>= 65 years	4	2.00	(12.83)	1	5.00	(NA)	0.73 [−52.72, 54.18]
Polaprezinc 150 mg							
< 65 years	61	11.34	(13.70)	80	2.29	(7.03)	8.87 [5.36, 12.38]
>= 65 years	7	15.00	(16.87)	7	3.00	(7.53)	8.79 [−9.20, 26.77]
Polaprezinc 300 mg							
< 65 years	15	21.87	(17.37)	9	6.33	(7.97)	22.03 [7.60, 36.45]
>= 65 years	4	25.50	(12.61)	2	2.50	(3.54)	30.00 [−75.39, 135.39]
**Comorbidities**							
Polaprezinc 75 mg							
No	6	3.00	(5.83)	1	10.00	(NA)	−3.55 [−10.26, 3.15]
Yes	3	4.00	(14.93)	5	4.80	(9.12)	3.88 [−15.63, 23.40]
Polaprezinc 150 mg							
No	29	12.41	(10.60)	48	2.98	(6.86)	7.84 [4.06, 11.62]
Yes	39	11.21	(16.13)	39	1.56	(7.24)	9.84 [4.22, 15.47]
Polaprezinc 300 mg							
No	11	22.82	(16.83)	5	7.60	(6.27)	21.32 [0.15, 42.48]
Yes	8	22.38	(16.51)	6	4.00	(8.39)	24.17 [4.42, 43.92]
**Baseline zinc concentration**							
Polaprezinc 75 mg							
< 60μg/dL	5	7.60	(10.09)	5	9.00	(2.35)	−0.09 [−12.10, 11.92]
>= 60μg/dL	4	−2.00	(1.41)	1	−11.00	(NA)	9.00 [0.39, 17.61]
Polaprezinc 150 mg							
< 60μg/dL	25	12.48	(9.29)	28	4.75	(6.32)	8.41 [4.07, 12.76]
>= 60μg/dL	43	11.28	(16.15)	59	1.20	(7.11)	8.86 [4.05, 13.67]
Polaprezinc 300 mg							
< 60μg/dL	3	37.00	(19.08)	6	9.50	(2.43)	27.26 [4.03, 50.48]
>= 60μg/dL	16	19.94	(14.78)	5	1.00	(8.92)	21.54 [5.78, 37.31]

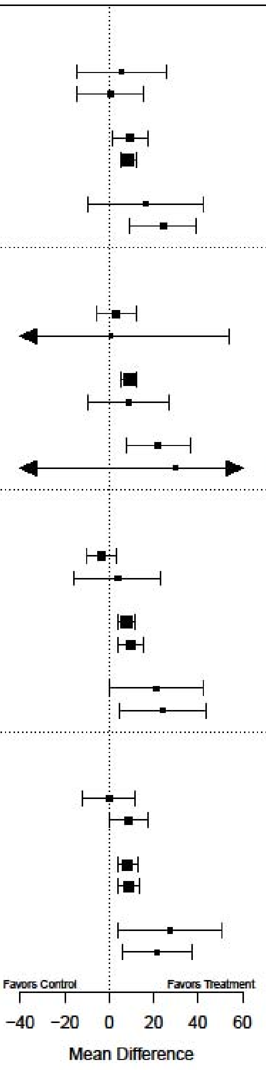

Figure S12. Subgroup analysis for changes from baseline in serum zinc concentration (μg/dL) (by dose of polaprezinc vs. placebo for primary analysis population, patients with serum zinc concentration of less than 70 μg/dL).

Table S1. Interaction effects of treatment by each covariate factor.

Treatment (vs. Placebo)	Item	Primary Analysis Population†			Secondary Analysis Population‡		
		Treatment-covariate interaction	(95% CI)	p-value	Treatment-covariate interaction	(95% CI)	p-value
Overall polaprezinc	SEX, Male (vs. Female)	-1.03	(-8.77, 6.71)	0.79	3.26	(-2.08, 8.60)	0.23
	AGE, per 10 years	0.13	(-2.66, 2.92)	0.92	-0.31	(-2.37, 1.75)	0.77
	Comorbidity, Yes (vs No)	1.85	(-5.42, 9.12)	0.62	2.94	(-2.44, 8.32)	0.28
	Baseline zinc concentration, per 10 µg/dL	0.49	(-6.20, 7.18)	0.89	-0.61	(-4.09, 2.87)	0.73
Polaprezinc 75mg/day	SEX, Male (vs. Female)	5.69	(-25.27, 36.64)	0.66	6.23	(-16.81, 29.27)	0.58
	AGE, per 10 years	5.64	(-10.77, 22.04)	0.42	3.47	(-2.80, 9.74)	0.26
	Comorbidity, Yes (vs No)	2.50	(-33.37, 38.36)	0.86	6.35	(-17.48, 30.18)	0.58
	Baseline zinc concentration, per 10 µg/dL	-15.35	(-43.59, 12.88)	0.22	-6.00	(-16.96, 4.96)	0.27
Polaprezinc 150mg/day	SEX, Male (vs. Female)	0.22	(-7.24, 7.69)	0.95	2.92	(-2.32, 8.16)	0.27
	AGE, per 10 years	1.27	(-1.54, 4.08)	0.37	-1.23	(-3.28, 0.82)	0.24
	Comorbidity, Yes (vs No)	0.92	(-6.26, 8.10)	0.80	1.92	(-3.32, 7.16)	0.47
	Baseline zinc concentration, per 10 µg/dL	1.29	(-5.22, 7.79)	0.70	1.07	(-2.36, 4.51)	0.54
Polaprezinc 300mg/day	SEX, Male (vs. Female)	-0.30	(-26.47, 25.88)	0.98	4.21	(-9.55, 17.97)	0.54
	AGE, per 10 years	1.80	(-9.47, 13.07)	0.74	5.59	(0.80, 10.38)	0.023
	Comorbidity, Yes (vs No)	4.22	(-28.30, 36.74)	0.79	3.28	(-10.54, 17.10)	0.64
	Baseline zinc concentration, per 10 µg/dL	-21.82	(-50.42, 6.78)	0.13	-11.42	(-19.62, -3.22)	0.007*

*: p<0.20, †: patients with serum zinc concentration of less than 70 µg/dL, ‡: patients with serum zinc concentration of less than 80 µg/dL

Table S2. Adverse drug reactions for primary analysis population, patients with serum zinc concentration of less than 70 μg/dL.

	Polaprezinc								Placebo	
	Overall (N=97)		75 mg/day (N=9)		150 mg/day (N=69)		300 mg/day (N=19)		(N=87)	
	no. of patients (%)									
All	18	(18.6)	2	(22.2)	10	(14.5)	6	(31.6)	13	(14.9)
Blood and lymphatic system disorders	1	(1.0)	0	(0.0)	1	(1.4)	0	(0.0)	0	(0.0)
Iron deficiency anaemia	1	(1.0)	0	(0.0)	1	(1.4)	0	(0.0)	0	(0.0)
Gastrointestinal disorders	6	(6.2)	0	(0.0)	2	(2.9)	4	(21.1)	2	(2.3)
Abdominal discomfort	1	(1.0)	0	(0.0)	0	(0.0)	1	(5.3)	1	(1.1)
Abdominal distension	2	(2.1)	0	(0.0)	1	(1.4)	1	(5.3)	0	(0.0)
Constipation	2	(2.1)	0	(0.0)	1	(1.4)	1	(5.3)	1	(1.1)
Dyspepsia	1	(1.0)	0	(0.0)	0	(0.0)	1	(5.3)	0	(0.0)
Nausea	1	(1.0)	0	(0.0)	0	(0.0)	1	(5.3)	0	(0.0)
Skin and subcutaneous tissue disorders	1	(1.0)	0	(0.0)	1	(1.4)	0	(0.0)	0	(0.0)
Eczema	1	(1.0)	0	(0.0)	1	(1.4)	0	(0.0)	0	(0.0)
Investigations	12	(12.4)	2	(22.2)	8	(11.6)	2	(10.5)	11	(12.6)
Blood copper increased	1	(1.0)	1	(11.1)	0	(0.0)	0	(0.0)	0	(0.0)
Blood iron decreased	6	(6.2)	1	(11.1)	4	(5.8)	1	(5.3)	4	(4.6)
Blood iron increased	0	(0.0)	0	(0.0)	0	(0.0)	0	(0.0)	1	(1.1)
Blood triglycerides increased	2	(2.1)	0	(0.0)	2	(2.9)	0	(0.0)	3	(3.4)
Blood urea increased	1	(1.0)	0	(0.0)	1	(1.4)	0	(0.0)	0	(0.0)
Blood uric acid increased	0	(0.0)	0	(0.0)	0	(0.0)	0	(0.0)	1	(1.1)
Eosinophil count increased	0	(0.0)	0	(0.0)	0	(0.0)	0	(0.0)	1	(1.1)
Gamma-glutamyltransferase increased	2	(2.1)	0	(0.0)	1	(1.4)	1	(5.3)	0	(0.0)
Haematocrit decreased	1	(1.0)	0	(0.0)	1	(1.4)	0	(0.0)	0	(0.0)
Haemoglobin decreased	1	(1.0)	0	(0.0)	1	(1.4)	0	(0.0)	0	(0.0)
Lymphocyte count decreased	0	(0.0)	0	(0.0)	0	(0.0)	0	(0.0)	1	(1.1)
Monocyte count increased	0	(0.0)	0	(0.0)	0	(0.0)	0	(0.0)	1	(1.1)
Red blood cell count decreased	1	(1.0)	0	(0.0)	1	(1.4)	0	(0.0)	0	(0.0)
White blood cell count increased	0	(0.0)	0	(0.0)	0	(0.0)	0	(0.0)	1	(1.1)
Blood alkaline phosphatase increased	1	(1.0)	1	(11.1)	0	(0.0)	0	(0.0)	0	(0.0)

MedDRA version (21.1)

Table S3. Adverse drug reactions for secondary analysis population, patients with serum zinc concentration of less than 80 μg/dL.

	Polaprezinc								Placebo	
	Overall (N=253)		75 mg/day (N=16)		150 mg/day (N=190)		300 mg/day (N=47)		(N=198)	
	no. of patients (%)									
All	37	(14.6)	2	(12.5)	21	(11.1)	14	(29.8)	29	(14.6)
Blood and lymphatic system disorders	1	(0.4)	0	(0.0)	1	(0.5)	0	(0.0)	0	(0.0)
Iron deficiency anaemia	1	(0.4)	0	(0.0)	1	(0.5)	0	(0.0)	0	(0.0)
Gastrointestinal disorders	11	(4.3)	0	(0.0)	4	(2.1)	7	(14.9)	6	(3.0)
Abdominal discomfort	2	(0.8)	0	(0.0)	1	(0.5)	1	(2.1)	2	(1.0)
Abdominal distension	3	(1.2)	0	(0.0)	1	(0.5)	2	(4.3)	1	(0.5)
Constipation	4	(1.6)	0	(0.0)	2	(1.1)	2	(4.3)	3	(1.5)
Dyspepsia	1	(0.4)	0	(0.0)	0	(0.0)	1	(2.1)	0	(0.0)
Nausea	1	(0.4)	0	(0.0)	0	(0.0)	1	(2.1)	0	(0.0)
Vomiting	1	(0.4)	0	(0.0)	0	(0.0)	1	(2.1)	0	(0.0)
Skin and subcutaneous tissue disorders	1	(0.4)	0	(0.0)	1	(0.5)	0	(0.0)	1	(0.5)
Alopecia	0	(0.0)	0	(0.0)	0	(0.0)	0	(0.0)	1	(0.5)
Eczema	1	(0.4)	0	(0.0)	1	(0.5)	0	(0.0)	0	(0.0)
Investigations	26	(10.3)	2	(12.5)	17	(8.9)	7	(14.9)	22	(11.1)
Alanine aminotransferase increased	3	(1.2)	0	(0.0)	2	(1.1)	1	(2.1)	0	(0.0)
Blood bilirubin increased	1	(0.4)	0	(0.0)	1	(0.5)	0	(0.0)	0	(0.0)
Blood cholesterol increased	0	(0.0)	0	(0.0)	0	(0.0)	0	(0.0)	1	(0.5)
Blood copper increased	1	(0.4)	1	(6.3)	0	(0.0)	0	(0.0)	0	(0.0)
Blood iron decreased	9	(3.6)	1	(6.3)	6	(3.2)	2	(4.3)	6	(3.0)
Blood iron increased	0	(0.0)	0	(0.0)	0	(0.0)	0	(0.0)	1	(0.5)
Blood triglycerides increased	8	(3.2)	0	(0.0)	5	(2.6)	3	(6.4)	6	(3.0)
Blood urea increased	1	(0.4)	0	(0.0)	1	(0.5)	0	(0.0)	1	(0.5)
Blood uric acid increased	0	(0.0)	0	(0.0)	0	(0.0)	0	(0.0)	1	(0.5)
Eosinophil count increased	2	(0.8)	0	(0.0)	2	(1.1)	0	(0.0)	1	(0.5)
Gamma-glutamyltransferase increased	2	(0.8)	0	(0.0)	1	(0.5)	1	(2.1)	0	(0.0)
Haematocrit decreased	1	(0.4)	0	(0.0)	1	(0.5)	0	(0.0)	0	(0.0)
Haemoglobin decreased	1	(0.4)	0	(0.0)	1	(0.5)	0	(0.0)	0	(0.0)
Lymphocyte count decreased	0	(0.0)	0	(0.0)	0	(0.0)	0	(0.0)	1	(0.5)
Monocyte count increased	0	(0.0)	0	(0.0)	0	(0.0)	0	(0.0)	1	(0.5)
Neutrophil count increased	0	(0.0)	0	(0.0)	0	(0.0)	0	(0.0)	1	(0.5)
Red blood cell count decreased	1	(0.4)	0	(0.0)	1	(0.5)	0	(0.0)	0	(0.0)
White blood cell count decreased	0	(0.0)	0	(0.0)	0	(0.0)	0	(0.0)	1	(0.5)
White blood cell count increased	0	(0.0)	0	(0.0)	0	(0.0)	0	(0.0)	2	(1.0)

Protein urine present	0	(0.0)	0	(0.0)	0	(0.0)	0	(0.0)	1	(0.5)
Blood alkaline phosphatase increased	2	(0.8)	1	(6.3)	0	(0.0)	1	(2.1)	1	(0.5)

MedDRA version (21.1)

Table S4. Serum concentration of copper and iron for secondary analysis population, patients with serum zinc concentration of less than 80 μg/dL

Time point	Treatment	Serum Copper Concentration (μg/dL)				Serum Iron Concentraiotn (μg/dL)			
		Mean ± SD	Min	Median	Max	Mean ± SD	Min	Median	Max
Baseline	Polaprezinc 75 mg/day	102.9 ± 17.0	71	105	134	107.3 ± 34.9	56	106.5	179
	Polaprezinc 150 mg/day	103.5 ± 20.7	37	102.0	193	91.2 ± 35.1	10	86.5	253
	Polaprezinc 300 mg/day	104.5 ± 18.2	73	103.0	152	90.2 ± 33.1	38	85.0	157
	Placebo	103.0 ± 18.2	62	101.0	169	88.1 ± 36.9	15	83.5	247
week 4	Polaprezinc 75 mg/day	107.6 ± 18.5	80	105.0	143	80.6 ± 37.4	20	75.0	175
	Polaprezinc 150 mg/day	100.9 ± 20.3	31	99.0	188	92.0 ± 41.1	18	86.0	319
	Polaprezinc 300 mg/day	101.2 ± 20.9	61	99.0	180	87.6 ± 31.4	24	83.0	175
	Placebo	103.4 ± 17.1	63	102.5	185	92.1 ± 40.5	11	87.0	250
week 8	Polaprezinc 75 mg/day	105.9 ± 14.6	81	107.0	142	86.1 ± 41.9	34	72.0	157
	Polaprezinc 150 mg/day	101.7 ± 21.2	36	99.5	182	87.8 ± 34.9	12	85.5	256
	Polaprezinc 300 mg/day	99.4 ± 19.7	69	97.0	184	82.8 ± 30.4	30	82.5	152
	Placebo	104.3 ± 18.4	67	103.0	167	88.5 ± 38.1	11	84.5	214
week 12	Polaprezinc 75 mg/day	105.8 ± 26.2	77	103.0	184	104.6 ± 56.7	43	98.5	240
	Polaprezinc 150 mg/day	101.8 ± 21.9	30	99.0	232	89.3 ± 33.9	12	89.0	191
	Polaprezinc 300 mg/day	100.2 ± 18.1	67	98.0	146	88.7 ± 38.3	25	80.5	185
	Placebo	104.2 ± 19.4	64	104.0	247	89.3 ± 35.2	22	86.0	249
